# In Search of Differences in the Perception of Safety Climate by Employees of an International Manufacturing Company

**DOI:** 10.3390/ijerph192214980

**Published:** 2022-11-14

**Authors:** Marta Znajmiecka, Elżbieta Roszko-Wójtowicz, Marta Stasiła-Sieradzka

**Affiliations:** 1 Institute of Psychology, University of Lodz, 90-001 Lodz, Poland; 2 Department of Economic and Social Statistics, University of Lodz, 90-214 Lodz, Poland; 3 Faculty of Social Sciences, Institute of Psychology, University of Silesia in Katowice, 40-126 Katowice, Poland

**Keywords:** safety culture, safety climate, measurement of safety climate, multinational manufacturing company in the construction industry, single-factor analysis of variance (ANOVA), Summary Safety Climate Indicator (SSCI)

## Abstract

The implementation of effective workforce safety programmes ought to be linked to an understanding of the specificity of the work in the organisation concerned, taking into consideration the assessment of the level of safety expressed by the professional group representing it at the executive level. The main purpose of the study presented in the paper, which is part of a broader project of researching safety culture in the organisation, is a diagnosis of the safety climate in the Polish branch of an international manufacturing company. The following research question was formulated: Is the examined international manufacturing company a homogeneous organisation from the point of view of assessing its safety culture? The research was exploratory. In total, 203 respondents, which amounts to 35% of the employees, participated in the study. The selection of the sample was representative—in proportion to the number of employees in individual departments and their positions in the examined organisation. The presented paper includes an analysis of the results obtained on the basis of the abbreviated version of the Safety Climate Questionnaire, a self-developed tool to assess ten separate dimensions of safety climate. The results of a single-factor analysis of variance (ANOVA) along with post hoc tests prove that there is a statistically significant difference between the respondents representing different positions in the organisation and different areas of employment. The position held in the company significantly differentiates the employees in a statistically significant way—in eight out of eleven diagnostic areas, including the Summary Safety Climate Indicator (SSCI). In the case of department, statistically significant differences were found in seven out of eleven diagnostic areas. Education proved to be the factor that differentiates the respondents the least in terms of the assessment of workplace safety climate. Statistically significant differences occurred only in three out of eleven diagnostic areas. The observed differences in the assessment of the dimensions of workplace safety climate point to the need for the promotion of more diversified and individualised measures, taking into account the specificity of work and the nature of hazards in a given position, and the creation of practical safety programmes not only in the procedural and technical dimensions but also in social and psychological ones.

## 1. Introduction

Workplace safety is an important objective from the point of view of both the organisation and the whole society [1,2]. Reducing accident rates is part of the overall trend of improving the quality of human life. The implementation of effective workplace safety programmes should be linked to an understanding of the specificity of the work in the organisation concerned, taking into account the assessment of the level of safety expressed by the professional group representing it at the executive level [3]. The aforementioned specificity of work is determined, among others, by the scope of subordination, individual vs. team task performance, the frequency of contact with technology, types of hazards occurring in the work environment, and the level of employee education. One of the important aspects of the analysis of the effectiveness of actions taken to improve safety is the study of workplace safety climate, which becomes the starting point for practical implementations. Such actions should be of a non-universal nature. Additionally, the verification of their effectiveness ought to be based on an adequate methodology for measuring the analysed phenomena [3,4].

The paper consists of four parts plus the introduction and conclusions. In the Introduction, the authors explain the need to conduct in-depth research in the area of safety culture in the workplace and present the structure of the paper. The theoretical part defines basic concepts and discusses the issue of safety climate as part of safety culture along with the studies published so far on the subject. The research goal, questions and hypothesis are formulated. The Material and Methods section presents the research tools used and the characteristics of the studied group. This section also describes the one-way analysis of variance (ANOVA) used for comparing means across multiple populations. The next section presents the results of empirical research carried out in 2021. The dimensions in which the presence of statistically significant differences was confirmed by post hoc tests in the group of surveyed employees are listed. In the Discussion, the authors compare the results of their own research with studies conducted by other researchers and indicate similarities and differences. In the Conclusions, the authors, among others, show some limitations of their own research and indicate the directions of further research in the area of safety culture. They also provide the practical implications of the research carried out.

## 2. Safety Climate as Part of Safety Culture in the Workplace

### 2.1. Theoretical Background

The evaluative perspective of safety culture aligns with the idea that culture is what an organisation should have to maintain safety—in particular, a fundamental commitment to the importance of safety and joint responsibility across all employees. Key contributions to safety culture as a concept include: (a) it is comprised of a fundamental sense of safety’s value and importance, which elevates safety issues above other competing priorities; (b) this value can be taught/conveyed to others either through specific human resource practices or leadership; and (c) it can be operationalised as a series of dimensions to reflect the values that organisational and leadership practices convey ([5]: p. 293). One of the basic conditions for an effective and desirable change in the level of workplace health and safety culture is, above all, its examination and description. Experts [5,6,7,8,9,10,11] dealing with the issue of diagnosing and shaping safety culture emphasise the possibility of taking into account the existence of two dimensions of safety culture in the conducted analyses. One is visible and related to safety management in the organisation Its analysis is associated with the assessment of the procedures used and the workplace itself in terms of its safety, inspections, causes and consequences of accidents, as well as the introduced remedial mechanisms. Diagnostic methods such as observations, study visits or audits can be used in this area. The other dimension is hidden, related to psychological factors, behaviour patterns, values, attitudes, and norms adopted in the organisation and their perception by employees. The study of this dimension involves employees and is not possible to implement only through the analysis of documentation. This hidden dimension is referred to as the safety climate of a given enterprise [12].

Workplace safety climate is thus seen as an area which allows for the identification of elements perceived by employees as problems in terms of occupational health and safety. D. Zohar [13] was the first to describe the safety climate in a company, treating it as an important element of the overall safety culture of this organisation. Using a questionnaire, he examined how employees of a given company perceived various aspects of the functioning of their workplace related to safety. The analysed dimensions included: the importance of OHS training, managers’ attitudes towards safety, the level of risk in the workplace, and the status of employees of OHS departments. To date, interviews and questionnaires have been the most commonly used tools to illustrate workplace safety climate.

Currently, many researchers [10,14,15,16,17] tend to define safety climate as a dimension of safety culture, its surface layer seen in the attitude of employees to various aspects of safety in the organisation. The safety climate study involves the employees themselves and cannot be carried out by external observers. It focuses on diagnosing how people perceive safety in their organisation, what they think and how they feel about it [18].

The safety climate study can be an element of benchmarking used in safety management [9,19] and become the basis for actions aimed to shape the desired attitudes and behaviours of employees. Benchmarking is focused on organisational learning and making creative use of others’ experiences. As emphasised by B. Karlof and S. Ostblom [20], benchmarking is a method that, by comparing with the best, allows us to determine whether the processes and functions carried out by the organisation should be improved. It enables the identification of factors thanks to which, while using the same procedures within the organisation, certain areas emerge where the expected effects are significantly better. As a result, data are obtained to initiate the process of improving the organisation and developing the skills of its leaders and rank-and-file employees. In comparative analyses, such factors as the industry, the size of the enterprise, the functioning of management systems and the employment of a person responsible for safety matters are important [21]. Years of service and a position held by an employee are also salient [20,21,22]. Many authors emphasise the need to study individual factors, such as age, gender or attitudes toward safety, pointing to their mediating role in the process of creating a safety culture [7,8,23,24,25]. Methodologies used to measure safety culture should therefore include combining qualitative and quantitative research, consisting of a ‘layered’ measurement of safety culture [4].

### 2.2. Aims and Hypotheses

The main purpose of the study was to diagnose the safety culture in the Polish branch of an international manufacturing company. The results presented in the paper are part of a broader research project on safety culture in the organisation in which the authors of the paper are involved. The following research question was formulated: is the examined international manufacturing company a homogeneous organisation from the point of view of assessing its safety culture? It was assumed that obtaining an answer to the research question would provide a basis for presenting recommendations for possible actions to promote best practices in each of the diagnosed areas of the organisation. Observing the lack of homogeneity within the distinguished comparison groups creates opportunities for developing appropriate actions in the technical, organisational and human resource areas, which in the future will allow for monitoring both the safety-related benefits and costs incurred. The research was exploratory, which is why the authors formulated the following non-directional hypothesis: the department where the person is employed, the position held and the level of education are factors differentiating the perception of safety culture by the employees of the analysed international manufacturing company. The non-directional hypothesis allows us to check the occurrence of differences between individual groups of employees, in a situation where we do not know for which group the results will be more favourable.

The applied methodology aimed at examining workplace safety culture was based on a triangulation approach. Qualitative measurement (focus interviews, workplace observations as well as an audit of safety and human resource management procedures) along with quantitative measurement (the Safety Climate Questionnaire and the Questionnaire Assessing Attitudes Towards Workplace Safety) were used [26,27]. The presented paper uses part of the results obtained in the course of the implementation of the above-mentioned research project which concerns the quantitative measurement of the workplace safety climate in this organisation.

### 2.3. Ethics Statements

The project was implemented in 2021. The approval for the study was granted by the Commission for Bioethics of Scientific Research of the University of Lodz (Personal safety climate level, RESOLUTION NO. 18/KBBN-UŁ/1/2020-21). All respondents provided written informed consent and anonymous data were kept confidential.

## 3. Materials and Methods

### 3.1. Data Description

**Figure 1:**
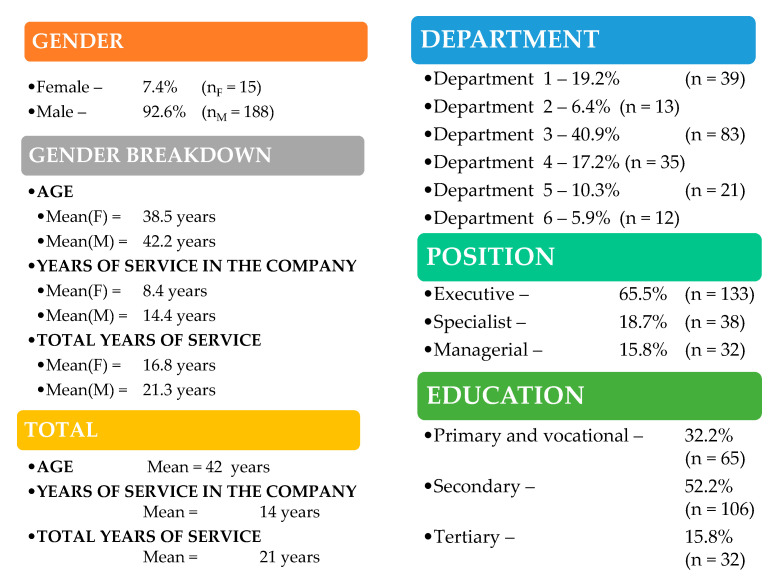
Selected measures of descriptive statistics for the surveyed population. Source: own elaboration based on the results of empirical research carried out in the analysed international manufacturing company, n = 203.

203 respondents participated in the survey, which accounts for 35% of the employees. The selection of the sample was representative of the level of the surveyed organisation. The respondents were selected in proportion to the number of employees in individual departments and positions in the studied organisation (taking into account the dimensions presented in Figure 1).

### 3.2. Research Tools

The study was carried out with the use of the paper and paper interviewing method. To measure safety climate, the Safety Climate Questionnaire (SCQ) by Znajmiecka-Sikora (see Appendix B), consisting of 50 statements, was used [27. The tool utilises the Likert scale, where 1 means strongly disagree, 2—disagree, 3—neither agree nor disagree, 4—agree, and 5—strongly agree. It allows us to diagnose safety climate by means of the Summary Safety Climate Indicator (SSCI), which consists of ten separate dimensions of safety climate, selected on the basis of theoretical analysis: (1) employee participation in safety-related matters (PA), (2) safe behaviour (SB), (3) management engagement in health and safety (ME), (4) modelling and enhancing safe behaviours in the organisation (MO), (5) workplace accident risk management (RB), (6) technical facilities and ergonomics (BO), (7) pace of work and fatigue level (WP), (8) occupational health and safety training process (TP), (9) atmosphere in the workplace (AT), and (10) organisational policy with regard to occupational health and safety management (PO) [28,29,30,31,32,33]. The content of individual statements was evaluated by independent qualified experts to assess the validity of the questionnaire, and the belonging of the survey items to individual scales and dimensions was confirmed during the exploratory factor analysis [34]. The reliability of the entire Safety Climate Questionnaire, calculated using the Cronbach alpha coefficient, is 0.93, and for individual scales of the questionnaire, it ranges from 0.68 to 0.89 [26]. The values that the SSCI can take range from 50 to 250, while each of the ten dimensions can take values from 5 to 25 [19,26]. The obtained result confirms the good diagnostic properties of the questionnaire. The value of the Cronbach Alpha coefficient for the data presented in the paper was also calculated, which for the SSCI was Alpha = 0.714. Determining the Alpha Cronbach coefficient on the basis of all ten dimensions and obtaining a result above 0.7 confirms that the construction of the summary index (SSCI) is not only substantively justified but also confirmed by the results of statistical analyses. This result means high consistency (reliability) of the scale [26].

### 3.3. Methods

In the empirical part, an attempt was made to answer the research question and test the formulated research hypothesis. The conducted analysis was carried out taking into account the following factors:

1. Department—Production: large items for the construction sector (Department 1), Production: small ceiling tiles for the construction sector (Department 2), Production: panels for the construction sector (Department 3), Maintenance (Department 4), Warehouse (Department 5), Laboratory (Department 6);
2. Position held—executive, specialist (engineering and technology, health and safety services), managerial (department manager, section manager);
3. Education—primary or vocational, secondary, and tertiary.

At the beginning of the study, it was verified whether the assumptions regarding the use of unifactorial analysis of variance were met: normality of the distribution of variables in each population (group) and homogeneity of variance in all populations (groups). Meeting the assumptions allowed us to use a one-factor analysis of variance (ANOVA) and to obtain the value of statistics F [35,36,37,38,39] (results—Appendix A—Table A1). Subsequently, it was determined which mean was significantly different from the others, which required the use of multiple-comparison tests (called post hoc tests) (results—Appendix A—Table A2). The fulfilled assumption about the equality of variance allows the use of, among others, the Bonferroni test for this purpose. The results of the analyses are presented in Appendix A Table A1 and Table A2, in which, due to its considerable length, only the results of multiple comparisons which turned out to be statistically significant are presented.

In a few cases, the assumption of homogeneity of variance was not met. In this situation, a robust test for equality of means—the Brown–Forsythe test—was used to assess the significance of the differences. Determining which mean was significantly different from the others required the use of multiple-comparison tests (called post hoc tests). The unfulfilled assumption about the equality of variance allows for the use of, among others, Tamhane’s T2 test for this purpose.

**Figure 2:**
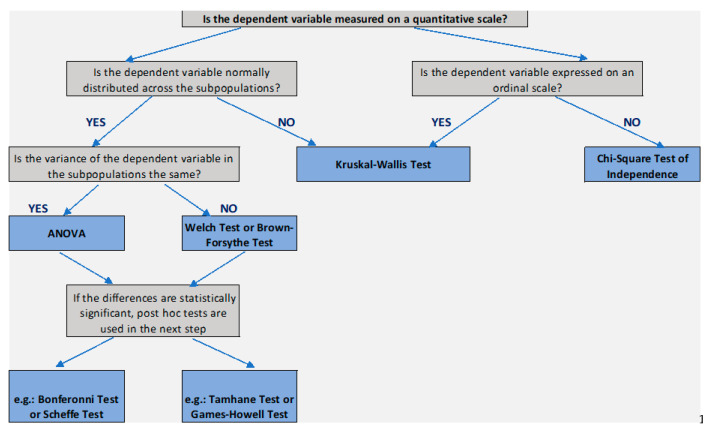
Diagram for selecting the appropriate test in the comparison of at least two populations—independent measurement. Source: own elaboration based on [36].

The procedure diagram is shown in Figure 2.

Additionally, the correlation between the employees’ age, the total length of service along with the years of service in the company and the individual dimensions of its safety culture, as well as the SSCI were assessed.

## 4. Results

### 4.1. Results Obtained from the Use of One-Way Analysis of Variance

**Figure 3:**
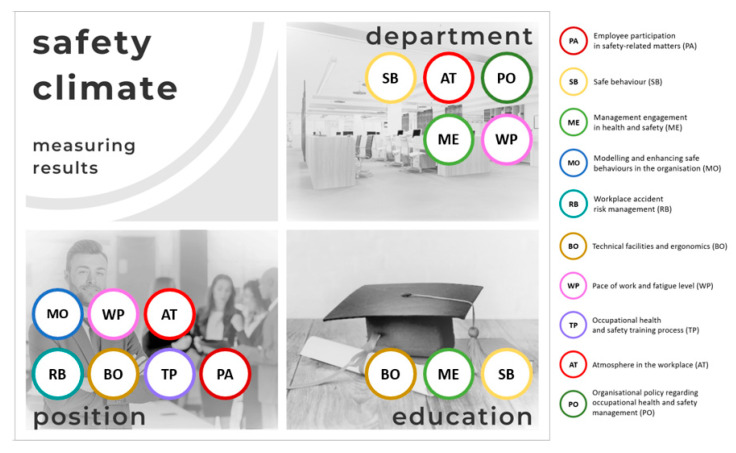
Statistically significant differences in the perception of safety culture dimensions broken down by social factors. Explanation: The dimensions of safety culture were assigned to individual social factors for which statistically significant results were obtained, confirmed subsequently by post hoc tests. Source: own elaboration based on the results of empirical research carried out in an international manufacturing company, n = 203.

The obtained results indicate that the position held in the company statistically significantly differentiates employees in as many as eight out of ten dimensions, and additionally in the SSCI. In the case of the department, statistically significant differences were observed in six out of ten dimensions and again in the SSCI. Education proved to be the least differentiating factor among the respondents in the area of assessing the safety climate in the workplace. Statistically significant differences occurred only in three out of ten dimensions (Figure 3, Appendix A Table A1 and Table A2). Thus, the formulated research hypothesis has been positively verified.

In addition, an in-depth statistical analysis based on the results of post hoc tests allowed for a clear indication of the dimensions in which, for individual social factors, the differences proved to be statistically significant (Figure 3, Appendix A Table A1 and Table A2).

In a detailed discussion of the results, it should be noted that statistically significant differences based on the department where a given employee works occur in relation to the Summary Safety Climate Indicator (SSCI) and dimensions of work safety climate such as safe behaviour (SB), management engagement in health and safety (ME), the atmosphere in the workplace (AT), pace of work and fatigue level (WP), as well as organisational policy with regard to occupational health and safety management (PO). The highest ratings are observed in the following departments: Production (Department 1), Maintenance (Department 4), and Warehouse (Department 5). The observed differences in the perception of safety culture among employees of individual departments are justified by the specificity of work. Production workers encounter the need to perform tasks in an unfavourable environment (e.g., noise), at the pace imposed by the machine, in a forced body position, with the use of numerous items of personal protective equipment (glasses, gloves, and specialised footwear). The workstations are associated with static loads or monotypic movements, which is related to the level of fatigue in the work performed. A lack of possibility or significantly reduced contact with other co-workers and a lack of teamwork resulting from the specificity of the workplace (mainly single-staff posts) significantly limit the opportunity for interpersonal contact with co-workers.

Belonging to the distinguished groups of employee positions statistically significantly differentiates the results in the field of safety culture assessment. The Summary Safety Climate Indicator (SSCI) shows the most favourable assessment on the part of the employees occupying specialist positions (Mspecialist = 189.55), followed by the employees occupying managerial positions (Mmanagerial = 187.72), with the lowest assessment declared by the employees in executive positions (Mexecutive = 182.12). This is undoubtedly related to the specificity of work performed in particular departments described above. Post hoc tests, however, showed statistically significant differences in the assessment of safety climate in relation to selected partial dimensions: workplace accident risk management (RB), technical facilities and ergonomics (BO), occupational health and safety training process (TP), employee participation in safety-related matters (PA), modelling and enhancing safe behaviours in the organisation (MO), the pace of work and fatigue level (WP), and atmosphere in the workplace (AT).

Education turned out to be the least differentiating factor among the respondents in terms of assessing workplace safety climate. Statistically significant differences were observed in relation to the following dimensions: safe behaviours (SB) and management engagement in health and safety (ME)—these dimensions are assessed most favourably by the employees with primary and vocational education (MSB = 17.11; MME = 17.17). Technical facilities and ergonomics (BO)are rated highest by the employees with tertiary education (MBO = 21.84).

### 4.2. Correlation Relationship Assessment

The next stage of the analysis was aimed at verifying whether there was a relationship between the perception of safety culture and age, total years of service and years of service in the company. The conducted analysis, based on the values of the Pearson linear correlation coefficient (Table 1), indicates that safe behaviours (SB), management engagement in health and safety (ME), organisational policy with regard to occupational health and safety management (PO), as well as the Summary Safety Climate Indicator were weakly and positively correlated with total years of service. In addition, the SSCI was positively correlated with years of service in the company. This means that as the length of service increases, the SSCI of workplace safety climate in the organisation increases as well.

## 5. Discussion

The obtained research results are consistent with previous studies of other researchers, presenting the difference in the assessment of workplace safety climate depending on the professional group [4,21,28,29]. Published research reports [30] indicate that safety climate is perceived differently by teams depending on the size of the company and the way of managing workplace safety, the gender of the respondents, the length of their service, the position held in the organisational structure, the ethnic origin, and the industry represented. Previous studies [3,7,30,31,32,33,34,35,36,37] indicate significant differences in the assessment of threats and the perception of safety climate presented by professional groups such as underground, open pit and drilling miners, ground airport personnel, sailors, people working in the polar regions, medical personnel, employees of the lighting and the construction industry, mountain rescuers, as well as employees of retail trade.

It is worth emphasising that so far differences in the perception of safety climate have been identified depending on the professional group in its so-called soft aspects (depending to a large extent on the competences of the managerial staff and the employees themselves), i.e., the modelling and strengthening of safe behaviours and atmosphere in the workplace. On the other hand, the so-called hard aspects (including standards and norms), i.e., technical facilities and ergonomics as well as the organisation’s policy in the field of health and safety, did not differentiate the analysed groups. This lack of differences in the assessment of the so-called hard areas related to the perception of safety culture is interpreted by the authors as a product of the basic area of activity of many organisations resulting from legal regulations as well as applicable international norms and standards. These actions are visible to employees since through the existing procedures they themselves are systematically included in such undertaken measures [3,29,38]. The presented analysis shows, however, that the perception of the organisation’s OHS policy varies depending on the length of service and the employment department. Additionally, the level of technical facilities and ergonomics is perceived differently by employees depending on their individual positions and level of education. This dimension is rated highest by people with tertiary education and employees of the following departments: Laboratory (6) and Maintenance (4), and lowest by Warehouse employees (5). This result has a practical meaning for the organisation—it means that there are different standards in the organisation in terms of safety management. On the other hand, the diverse perception in the field of technical facilities and ergonomics can be explained by the fact of expansion and modernisation of the enterprise—the newly created departments have a new machine park, and the workplaces meet the standards of modern ergonomics.

The position of the surveyed persons in the organisational structure is an important aspect as well. Research proves [39] that a higher position in the organisational structure, and thus a greater impact on the shaping of workplace safety culture in the organisation, is associated with a more positive assessment of its workplace safety climate. The results obtained by the authors do not confirm this thesis—the highest perception of safety culture occurs in the group of employees working in specialist positions.

The presented study shows that one organisation with its own safety standards (specific corporate standards set at the international level) is characterised by a different perception of safety climate depending on the specificity of work: the area of employment, position, education, and length of service, which means that it does not constitute a homogeneous environment in this respect. The revealed diversity of assessments of workplace safety climate implies the need to take practical actions aimed at occupational safety, not only in the procedural and technical area but also taking into account its other dimensions, i.e., social and psychological ones. It can be the basis for creating and implementing preventive programmes designed adequately to the diagnosed specificity and aimed at strengthening those aspects that require corrective actions [2,28]. These recommendations may concern both training and other forms of impact, e.g., shaping attitudes through modelling and providing appropriate role models by leaders, the scope of execution of programmes implemented by the OHS and HR Departments, as well as the scope and method of communication [7,40]. The results of systematically conducted research may form the basis for comparisons between the specified units within a given organisation or between organisations. This would enable the selection of safety leaders and the most effective practices within the organisation itself.

Many authors emphasise [10,36,37,41,42,43,44] that the study of safety climate can be considered as a basis for developing practical recommendations for improving workplace safety in an organisation. Recommendations relate primarily to the spheres of human activity such as shaping standards and values, safety education, building a sense of community and co-responsibility for others, responsible management and authority building, as well as motivating for safe behaviours. The implementation of programmes for modifying hazardous behaviours based on the safety climate study contributes to the improvement of the safety culture and quality of life of employees and is an effective tool in accident prevention.

## 6. Conclusions

The conducted analysis indicates the lack of homogeneity of the organisation in the perception of its safety climate depending on the position held, the area of employment, education, age and length of service. The observed differences in the assessment of the studied dimensions of workplace safety climate lead to the promotion of additional actions undertaken in a more individualised way which takes into account the specificity of work and the nature of hazards occurring in the work environment of representatives of various positions and departments. The revealed differences in the assessment of workplace safety climate thus become not only the reason but also the basis for work on practical implementations of measures aimed at improving safety not only in the procedural and technical dimension but also taking into account other possible diagnostic dimensions [10,44].

We trust that the presented results may become the basis for the development of programmes for modifying hazardous behaviours, based on workplace safety climate studies, which can be implemented in practice. Such programmes could contribute to improving the safety culture and quality of life of employees. For this reason, the results of the conducted research are particularly useful for managers and specialists dealing with human resources consulting, health and safety services or occupational psychologists.

The research presented in this paper did not take into account individual factors related to personality traits or other internal predispositions of individuals performing a given job. We believe that it would be desirable to supplement the research model with selected psychological features. Combining these factors into one coherent model would allow us to build a more complete picture of the relevant determinants of the assessment of workplace safety climate in an organisation.

At this stage of the study, the authors did not have the opportunity to discuss differences in the perception of safety culture based on gender, as a group of women had a small share of the studied population. Although gender is an important factor differentiating the perception of workplace safety climate, in this study, it was not possible to assess these differences. The percentage of women in the study population was too low.

## Figures and Tables

**Table 1:** Assessment of correlation relationship for selected diagnostic variables in individual dimensions of safety climate.

Dimensions/Type of Comparisons		PA	SB	ME	MO	RB	BO	WP	TP	AT	PO	SSCI
Age	r	0.050	0.066	0.119	0.036	0.087	0.080	−0.037	0.098	0.121	0.114	0.127
	p	0.481	0.348	0.091	0.612	0.216	0.256	0.599	0.163	0.087	0.104	0.071
Total years of service	r	0.020	0.164 *	0.196 **	−0.003	0.064	0.001	0.055	0.024	0.126	0.142 *	0.169 *
	p	0.777	0.019	0.005	0.967	0.361	0.984	0.435	0.729	0.073	0.044	0.016
Years of service in the company	r	0.020	0.164	0.196	−0.003	0.064	0.001	0.055	0.024	0.126	0.142	0.169 *
	p	0.777	0.019	0.005	0.967	0.361	0.984	0.435	0.729	0.073	0.044	0.016

Explanation: ** Correlation is significant at the 0.01 level (two-tailed). * Correlation is significant at the 0.05 level (two-tailed). Source: own elaboration based on the results of empirical research carried out in an international manufacturing company, n = 203.

## Data Availability

Data in aggregated form are presented in this paper.

## Appendix A

**Table A1:** The results of a single-factor analysis of variance ANOVA.

Dimensions		PA		SB		ME		MO		RB		BO		WP		TP		AT		PO		SSCI	
Factors																							
		**Department**																					
Department 1	M	18.67	F = 1.353	20.15	**F = 94.768**	20.28	**F = 94.037**	19.41	F = 0.781	19.51	B-F = 0.680	20.21	**F = 2.614**	16.72	**B-F = 3.276**	20.00	F = 1.421	20.paź	**B-F = 21.770**	19.97	**B-F = 5.265**	195.03	**B-F = 22.128**
	Me	19.00	*p* = 0.244	20.00	***p*** **< 0.001**	20.00	***p*** **< 0.001**	20.00	*p* = 0.565	19.00	*p* = 0.640	20.00	***p*** **= 0.026**	17.00	***p*** **= 0.010**	20.00	*p* = 0.218	20.00	***p*** **< 0.001**	20.00	***p*** **< 0.001**	195.00	***p*** **< 0.001**
	Sd	2.309		2.641		3.043		2.099		1.745		2.203		2.350		1.806		1.429		1.693		13.529	
Department 2	M	17.38	df_A_ = 5	10.69	df_A_ = 5	11.00	df_A_ = 5	19.31	df_A_ = 5	19.92	df_1_= 5	20.69	df_A_ = 5	17.31	df_1_= 5	19.23	df_A_ = 5	18.46	df_1_= 5	18.23	df_1_= 5	172.23	df_1_= 5
	Me	17.00	df_R_ = 197	10.00	df_R_ = 197	11.00	df_R_ = 197	20.00	df_R_ = 197	20.00	df_2_= 75.852	20.00	df_R_ = 197	17.00	df_2_= 71.806	20.00	df_R_ = 197	19.00	df_2_= 84.895	18.00	df_2_= 107.017	172.00	df_2_= 84.317
	Sd	3.150		2.594		1.633		1.974		3.040		2.213		4.906		2.619		1.761		1.166		7.201	
Department 3	M	18.63		11.95		11.49		19.92		19.41		20.84		14.87		20.43		18.34		18.59		174.47	
	Me	19.00		12.00		11.00		20.00		20.00		21.00		15.00		20.00		18.00		18.00		173.00	
	Sd	3.345		2.971		3.270		2.746		2.664		2.472		3.530		2.176		1.727		1.881		11.919	
Department 4	M	18.54		20.34		21.20		19.66		19.74		21.51		14.69		20.77		22.09		20.00		198.54	
	Me	18.00		21.00		21.00		20.00		20.00		21.00		15.00		20.00		22.00		20.00		197.00	
	Sd	3.221		3.077		2.898		2.485		2.214		2.228		3.513		1.987		2.049		2.236		16.750	
Department 5	M	17.29		20.76		20.14		19.paź		18.62		19.62		17.19		20.14		21.00		18.81		192.67	
	Me	18.00		20.00		20.00		20.00		20.00		20.00		16.00		20.00		21.00		20.00		196.00	
	Sd	2.901		2.737		3.705		2.256		3.500		2.247		3.932		2.372		2.915		2.482		22.397	
Department 6	M	19.58		11.00		10.00		20.50		20.17		21.75		14.58		20.92		18.92		18.50		175.92	
	Me	19.50		11.00		10.00		20.00		19.00		21.00		15.00		20.50		19.00		18.00		174.00	
	Sd	2.778		2.089		1.859		2.908		3.070		2.454		3.343		2.353		1.443		1.732		11.000	
		**Education**																					
Primary or vocational	M	18.23	F = 0.311	17.lis	**F = 3.499**	17.17	**B-F = 3.803**	19.71	F = 0.042	19.15	F = 1.185	20.32	**F = 4.588**	16.03	F = 1.210	20.20	F = 0.655	19.75	F = 0.963	19.08	F = 0.164	186.75	F = 1.477
	Me	18.00	*p* = 0.733	18.00	***p*** **= 0.032**	18.00	***p*** **= 0.026**	20.00	*p* = 0.959	19.00	*p* = 0.308	20.00	***p*** **= 0.011**	16.00	*p* = 0.300	20.00	*p* = 0.521	20.00	*p* = 0.384	19.00	*p* = 0.849	185.00	*p* = 0.231
	Sd	3.166		5.084		4.955		2.596		2.195		2.568		3.377		2.230		2.616		2.273		20.165	
Secondary	M	18.52	df_A_ = 2	14.99	df_A_ = 2	14.69	df_1_= 2	19.64	df_A_ = 2	19.53	df_A_ = 2	20.69	df_A_ = 2	15.51	df_A_ = 2	20.29	df_A_ = 2	19.44	df_A_ = 2	19.05	df_A_ = 2	182.35	df_A_ = 2
	Me	19.00	df_R_ = 202	14.00	df_R_ = 202	12.00	df_2_= 98.274	20.00	df_R_ = 202	20.00	df_R_ = 202	20.00	df_R_ = 202	16.00	df_R_ = 202	20.00	df_R_ = 202	19.00	df_R_ = 202	19.00	df_R_ = 202	180.00	df_R_ = 202
	Sd	2.859		5.070		5.549		2.435		2.822		2.257		3.576		2.056		2.103		1.884		16.488	
Tertiary	M	18.72		15.50		15.63		19.78		20.00		21.84		14.84		20.72		20.06		19.28		186.38	
	Me	20.00		15.50		12.00		20.00		20.00		22.00		15.00		20.00		20.00		19.50		184.00	
	Sd	3.603		5.316		6.333		2.587		2.476		2.157		3.993		2.317		2.564		2.067		16.502	
		**Position held**																					
Executive	M	18.08	**F = 3.811**	15.69	F = 2.805	15.43	B-F = 2.409	19.33	**F = 5.710**	19.05	**F = 12.722**	20.32	**F = 8.208**	16.08	**F = 4.671**	19.95	**F = 8.286**	19.32	**F = 5.584**	18.87	F-2.357	182.12	**F = 3.317**
	Me	18.00	***p*** **= 0.024**	15.00	*p* = 0.063	14.00	*p* = 0.096	20.00	***p*** **= 0.004**	19.00	***p*** **< 0.001**	20.00	***p*** **< 0.001**	16.00	***p*** **= 0.010**	20.00	***p*** **< 0.001**	19.00	***p*** **= 0.004**	19.00	*p* = 0.097	178.00	***p*** **= 0.038**
	Sd	3.060		4.945		5.178		2.470		2.504		2.375		3.599		2.119		2.176		2.009		17.682	
Specialist	M	18.74	df_A_ = 2	17.18	df_A_ = 2	17.39	df_1_= 2	19.87	df_A_ = 2	19.34	df_A_ = 2	21.18	df_A_ = 2	15.03	df_A_ = 2	20.63	df_A_ = 2	20.74	df_A_ = 2	19.45	df_A_ = 2	189.55	df_A_ = 2
	Me	19.00	df_R_ = 202	18.50	df_R_ = 202	20.00	df_2_= 86.862	20.00	df_R_ = 202	19.50	df_R_ = 202	21.00	df_R_ = 202	15.00	df_R_ = 202	20.00	df_R_ = 202	21.00	df_R_ = 202	20.00	df_R_ = 202	188.00	df_R_ = 202
	Sd	2.806		5.260		6.083		2.792		2.386		2.154		2.973		1.731		2.457		2.089		17.597	
Managerial	M	19.69		14.28		14.38		20.94		21.47		22.06		14.09		21.56		19.66		19.59		187.72	
	Me	19.50		12.00		11.50		21.00		22.00		22.00		14.00		22.00		19.50		20.00		185.00	
	Sd	3.167		5.715		6.257		1.795		2.272		2.184		3.788		2.271		2.598		1.998		17.190	

Note: All statistically significant differences are bolded. Explanations for the abbreviations used: M—mean, Me—median, Sd—standard deviation, df—number of the degrees of freedom, F—F statistic, B-F—Brown–Forsythe test. Source: own elaboration based on the results of empirical research carried out in an international manufacturing company, n = 203.

**Table A2:** The results of post hoc tests.

Department							
Dimensions	SB	ME	WP	AT	PO	SSCI	
Department 1	Statistically significant differences between Department 1 and Department 2 (*p* < 0.001), Department 3 (*p* < 0.001) and Department 6 (*p* < 0.001)	Statistically significant differences between Department 1 and Department 2 (*p* < 0.001), Department 3 (*p* < 0.001) and Department 6 (*p* < 0.001)	Statistically significant differences between Department 1 and Department 3 (*p* = 0.013)	Statistically significant differences between Department 1 and Department 3 (*p* < 0.001) and Department 4 (*p* < 0.001)	Statistically significant differences between Department 1 and Department 2 (*p* = 0.004) and Department 3 (*p* = 0.002)	Statistically significant differences between Department 1 and Department 2 (*p* < 0.001), Department 3 (*p* < 0.001) and Department 6 (*p* < 0.001)	
Department 2	Statistically significant differences between Department 2 and Department 1 (*p* < 0.001), Department 4 (*p* < 0.001) and Department 5 (*p* < 0.001)	Statistically significant differences between Department 2 and Department 1 (*p* < 0.001), Department 4 (*p* < 0.001) and Department 5 (*p* < 0.001)		Statistically significant differences between Department 2 and Department 4 (*p* < 0.001) and Department 5 (*p* = 0.050)	Statistically significant differences between Department 2 and Department 1 (*p* = 0.004) and Department 4 (*p* = 0.014)	Statistically significant differences between Department 2 and Department 1 (*p* < 0.001), Department 4 (*p* < 0.001) and Department 5 (*p* = 0.010)	
Department 3	Statistically significant differences between Department 3 and Department 1 (*p* < 0.001), Department 4 (*p* < 0.001) and Department 5 (*p* < 0.001)	Statistically significant differences between Department 3 and Department 1 (*p* < 0.001), Department 4 (*p* < 0.001) and Department 5 (*p* < 0.001)	Statistically significant differences between Department 3 and Department 1 (*p* = 0.013)	Statistically significant differences between Department 3 and Department 1 (*p* < 0.001), Department 4 (*p* < 0.001) and Department 5 (*p* = 0.008)	Statistically significant differences between Department 3 and Department 1 (*p* = 0.002) and Department 4 (*p* = 0.027)	Statistically significant differences between Department 3 and Department 1 (*p* < 0.001), Department 4 (*p* < 0.001) and Department 5 (*p* = 0.023)	
Department 2	Statistically significant differences between Department 4 and Department 2 (*p* < 0.001), Department 3 (*p* < 0.001) and Department 6 (*p* < 0.001)	Statistically significant differences between Department 4 and Department 2 (*p* < 0.001), Department 3 (*p* < 0.001) and Department 6 (*p* < 0.001)		Statistically significant differences between Department 4 and Department 1 (*p* < 0.001), Department 2 (*p* < 0.001), Department 3 (*p* < 0.001) and Department 6 (*p* < 0.001)	Statistically significant differences between Department 4 and Department 2 (*p* = 0.014) and Department 3 (*p* = 0.027)	Statistically significant differences between Department 4 and Department 2 (*p* < 0.001), Department 3 (*p* < 0.001) and Department 6 (*p* < 0.001)	
Department 5	Statistically significant differences between Department 5 and Department 2 (*p* < 0.001), Department 3 (*p* < 0.001) and Department 6 (*p* < 0.001)	Statistically significant differences between Department 5 and Department 2 (*p* < 0.001), Department 3 (*p* < 0.001) and Department 6 (*p* < 0.001)		Statistically significant differences between Department 5 and Department 2 (*p* = 0.050) and Department 3 (*p* = 0.008)		Statistically significant differences between Department 5 and Department 2 (*p* = 0.010) and Department 3 (*p* = 0.023)	
Department 6	Statistically significant differences between Department 6 and Department 1 (*p* < 0.001), Department 4 (*p* < 0.001) and Department 5 (*p* < 0.001)	Statistically significant differences between Department 6 and Department 1 (*p* < 0.001), Department 4 (*p* < 0.001) and Department 5 (*p* < 0.001)		Statistically significant differences between Department 6 and Department 4 (*p* < 0.001)		Statistically significant differences between Department 6 and Department 1 (*p* < 0.001) and Department 4 (*p* < 0.001)	
**Education**							
**Dimensions**	**SB**	**ME**	**BO**				
Primary or vocational	Statistically significant differences between primary or vocational and secondary education (*p* = 0.028)	Statistically significant differences between primary or vocational and secondary education (*p* = 0.009)	Statistically significant differences between primary or vocational and tertiary education (*p* = 0.009)				
Secondary	Statistically significant differences between primary or vocational and secondary education (*p* = 0.028)	Statistically significant differences between primary or vocational and secondary education (*p* = 0.009)					
Tertiary			Statistically significant differences between tertiary and primary or vocational (*p* = 0.009)				
**Position held**							
**Dimensions**	**PA**	**MO**	**RB**	**BO**	**WP**	**TP**	**AT**
Executive	Statistically significant differences between Executive and Managerial (*p* = 0.023)	Statistically significant differences between Executive and Managerial (*p* = 0.003)	Statistically significant differences between Executive and Managerial (*p* < 0.001)	Statistically significant differences between Executive and Managerial (*p* < 0.001)	Statistically significant differences between Executive and Managerial (*p* = 0.014)	Statistically significant differences between Executive and Managerial (*p* < 0.001)	Statistically significant differences between Executive and Specialist (*p* = 0.003)
Specialist			Statistically significant differences between Specialist and Managerial (*p* = 0.001)				Statistically significant differences between Specialist and Executive (*p* = 0.003)
Managerial	Statistically significant differences between Managerial and Executive (*p* = 0.023)	Statistically significant differences between Managerial and Executive (*p* = 0.003)	Statistically significant differences between Managerial and Executive (*p* < 0.001) and Specialist (*p* = 0.001)	Statistically significant differences between Managerial and Executive (*p* < 0.001)	Statistically significant differences between Managerial and Executive (*p* = 0.014)	Statistically significant differences between Managerial and Executive (*p* < 0.001)	

Note 1: Figure 1 and Table 1 should be analysed together. This will not only allow for the proper identification of statistically significant differences (Figure 1 and Table 1) but will also allow for the indication which groups assigned a higher rating to which dimensions of the organisation’s safety culture (Figure 1). Note 2: Individual social factors were assigned dimensions of safety culture for which statistically significant results were obtained, confirmed by post hoc tests. Note 3: Explanation to the background colours used in the table. Source: own elaboration based on the results of empirical research carried out in an international manufacturing company, n = 203.

## Appendix B. Safety Climate Questionnaire (SCQ)—Short Version

*Instructions:*

*This questionnaire contains statements which present human behaviour in various occupational situations and work institutions. Read each statement carefully and circle the answers to show the extent to which the given statement is true for you (based on your work experience).*

*Circle **SA**—if you **strongly agree**; A—if you **agree**, **NAND**—if you **neither agree nor disagree**; **DA**—if you **disagree**, **SDA**—if you **strongly disagree**.*

*Please answer as if your objective were to give a reliable description of your behaviour at work, the behaviour of your superiors, and work organisation.*

*No.*	*question*	*replies:*
		*SA – strongly agree*
		*A – agree*
		*NANDA – neither agree nor disagree*
		*DA – disagree*
		*SDA – strongly disagree*
1.	The machines and devices I operate undergo interim check-ups.	SA A NANDA DA SDA
2.	I have been involved in the occupational risk assessment process at my workstation.	SA A NANDA DA SDA
3.	The employer’s actions linked to occupational safety are consulted with employees.	SA A NANDA DA SDA
4.	From time to time, I happen to perform my duties not in compliance with the rules of occupational safety and health.	SA A NANDA DA SDA
5.	Employees can test personal protection equipment (e.g., footwear, goggles and glasses) prior to the decision to purchase them.	SA A NANDA DA SDA
6.	In our firm employees are members of the teams working to improve safety within the organisation (*e.g., they participate in formulating safety procedures, OHS instructions, in the works of post-accident teams, risk assessment, OSH committee*).	SA A NANDA DA SDA
7.	My workstation is orderly and tidy (tools are kept in the same place and waste is removed on a current basis) and it is important for me to keep the workstation tidy.	SA A NANDA DA SDA
8.	Employees are advised of any implementation of long-term safety projects (*e.g., ISO, OHSAS, prophylactic programmes, behaviours at the workstation monitoring programme*).	SA A NANDA DA SDA
9.	My employer takes measures to improve safety within the organisation.	SA A NANDA DA SDA
10.	My superiors are not interested in OSH issues.	SA A NANDA DA SDA
11.	From time to time my superior happens to assign a task to be fulfilled contrary to the rules of occupational safety (*e.g., to remove the protective shield from the machine so that it works faster*).	SA A NANDA DA SDA
12.	My superior intervenes whenever the work safety rules are being breached.	SA A NANDA DA SDA
13.	I disregard my superior’s remarks and instructions on safety (*e.g., I do not wear protective gloves, footwear, hearing protectors, even though my superior has admonished me to wear them*).	SA A NANDA DA SDA
14.	From time to time, I happen to behave riskily (*e.g., I remove protective shields from machines, carry out minor repairs while the machine is in operation, exceed the allowable speed, take shortcuts to hit the target more quickly*).	SA A NANDA DA SDA
15.	The plant has an ‘accidents at work’ information system (*e.g., a mannequin with locations of injuries, information board, newsletter*).	SA A NANDA DA SDA
16.	In my firm, safe work performance by employees is promoted (*e.g., by being taken into account in the employee assessment process*).	SA A NANDA DA SDA
17.	From time to time, my superior happens not to follow the safety rules.	SA A NANDA DA SDA
18.	The number of tasks I have to perform on a daily basis causes me to have to work at a very fast pace.	SA A NANDA DA SDA
19.	My superior gives me feedback on my work performance, bringing attention to the fact that I must work safely.	SA A NANDA DA SDA
20.	My superior’s conduct sets an example for me in terms of safety.	SA A NANDA DA SDA
21.	My superiors actively participate in safety promotion actions organised by the firm.	SA A NANDA DA SDA
22.	Each and every accident that has happened in our plant is discussed by the superior during information meetings.	SA A NANDA DA SDA
23.	The plant maintains a logbook of potentially dangerous incidents (near-accidents).	SA A NANDA DA SDA
24.	The logbook of potentially dangerous incidents (near-accidents) is used, for example, to advise employees of the risks and prophylactic measures taken.	SA A NANDA DA SDA
25.	My superiors ‘turn a blind eye’ on how work is performed—no matter whether or not it is done safely. What matters is timeliness and the required output.	SA A NANDA DA SDA
26.	Particularly dangerous places are adequately marked.	SA A NANDA DA SDA
27.	I usually work under time pressure.	SA A NANDA DA SDA
28.	Our organisation has an action plan to improve occupational safety.	SA A NANDA DA SDA
29.	The work I do is very strenuous for me.	SA A NANDA DA SDA
30.	My daily work schedule changes very often.	SA A NANDA DA SDA
31.	I feel that my superior motivates me to work safely.	SA A NANDA DA SDA
32.	After the entire day of work, I suffer from various muscular/backbone ailments (*e.g., corns, backbone pains*).	SA A NANDA DA SDA
33.	The rules of moving around the plant are clearly specified.	SA A NANDA DA SDA
34.	Machines and devices are repaired by qualified teams.	SA A NANDA DA SDA
35.	Control elements of machines are well visible and marked.	SA A NANDA DA SDA
36.	I have been advised of the occupational risk assessment at my workstation.	SA A NANDA DA SDA
37.	I have a good relationship with my superior and I know I can rely on him/her in case of an emergency.	SA A NANDA DA SDA
38.	When doing a dangerous job, I know I can trust my colleagues with whom I am fulfilling a dangerous task.	SA A NANDA DA SDA
39.	I feel well-informed about protection against my occupational risks.	SA A NANDA DA SDA
40.	I believe I am well-trained in providing first aid and if an accident were to happen, I would know what to do.	SA A NANDA DA SDA
41.	I know the employee and the employer’s duties in respect of safety.	SA A NANDA DA SDA
42.	Upon change of my workstation—prior to starting work at a new workstation I am given the workstation instruction.	SA A NANDA DA SDA
43.	I have a good relationship with my colleagues in the workplace.	SA A NANDA DA SDA
44.	In our organisation safety is a priority, the process of improving safety is continuous.	SA A NANDA DA SDA
45.	Communication within the team is difficult, there is no knowing who you can trust.	SA A NANDA DA SDA
46.	Our organisation continuously takes various measures to improve work safety.	SA A NANDA DA SDA
47.	In our organisation, safety issues are only discussed when an accident or inspection takes place.	SA A NANDA DA SDA
48.	Our firm conducts practical exercises (*e.g., evacuation exercise, accident simulation*).	SA A NANDA DA SDA
49.	When working in a team, I know I can trust the other team members.	SA A NANDA DA SDA
50.	When doing work I know very well, I happen to flout the OSH rules from time to time.	SA A NANDA DA SDA

*Please check if you have answered all the questions!*
